# Segmental vertebral artery agenesis with deep cervical reconstitution in a cervicothoracic variant cluster

**DOI:** 10.1007/s00276-026-03937-4

**Published:** 2026-07-06

**Authors:** Mugurel Constantin Rusu, Victor Ioan Tibacu, Bogdan Vintilă, Vladimir Ioan Zamfirescu

**Affiliations:** Division of Anatomy, Faculty of Dentistry, “Carol Davila” University of Medicine and Pharmacy, 050474 Bucharest, Romania

**Keywords:** Common brachiocephalic–left common carotid origin, Cervical ribs, Vertebral artery agenesis, Deep cervical artery

## Abstract

**Purpose:**

To report a complex cervicothoracic vascular-osseous phenotype detected on CTA and define its surgical and radiological relevance.

**Methods:**

Neck CTA of a 76-year-old woman was reviewed on multiplanar reconstructions for aortic arch branching, supraaortic courses, vertebral artery segments, collateral channels, cervical ribs, styloid processes, and pharyngeal carotid relationships.

**Results:**

CTA showed a common origin of the brachiocephalic trunk and left common carotid artery (colloquially termed bovine aortic arch), bilateral rudimentary cervical ribs, high-riding pretracheal brachiocephalic trunk, and marked supraaortic tortuosity. The brachiocephalic trunk reached C7 and divided low in the neck below the right thyroid lobe. The right common carotid artery formed a proximal loop, and the right vertebral artery arose posteriorly from the right subclavian artery, passed anterior to the right cervical rib, and entered the C6 transverse foramen. On the left, the common carotid artery formed a 2.12-cm posterolateral arch loop. The left vertebral ostium, V1 segment, and proximal V2 segment were absent. A tortuous left deep cervical artery arose from the left subclavian artery, passed anterior to the left cervical rib, and reconstituted the distal V2 segment at the C3–C4 disc level; the distal artery continued as a PICA-type vertebral artery. Additional findings were a medial loop of the right external carotid artery, a 1.56-cm retropharyngeal/retrotonsillar loop of the right internal carotid artery, bilateral elongated styloid processes, and bilateral poor sternoclavicular joint articulations with the left brachiocephalic vein partly coursing in the resulting interclavicular space.

**Conclusion:**

The case illustrates interacting cervicothoracic variants that may affect anterior neck surgery, thyroid and pharyngeal procedures, tracheostomy, supraclavicular exposure, endovascular access, and CTA interpretation.

## Introduction

The cervicothoracic junction is a topographically constrained corridor through which the major supraaortic trunks, vertebral arteries, and their branches converge before entering the neck. Anatomical variants of the aortic arch branching pattern, supraaortic courses, and vertebral artery segments are well recognised in the radiological and surgical literature but are most often reported in isolation. The clinical significance of such variants is greatest when they interact, because the compound effect on the operative corridor may exceed the sum of the individual anomalies.

The common brachiocephalic–left common carotid origin (so-called bovine aortic arch)—defined as a common origin of the brachiocephalic trunk (BCT) and the left common carotid artery from the aortic arch—is the most prevalent branching variant of the human aortic arch. The term “bovine aortic arch” is an established but anatomically inaccurate misnomer, as this configuration does not correspond to the branching pattern of the bovine aortic arch; the descriptive designation “common brachiocephalic–left common carotid origin” is therefore preferred and is used hereafter, with “bovine arch” retained only as the colloquial label [12]. Rotundu et al. [11] reviewed its medical and surgical implications and confirmed its relevance to open and endovascular procedures, including the heightened risk of aortic dissection entry in the aortic arch and the navigational constraints it imposes during catheter-based interventions. When this common origin coexists with a high-riding brachiocephalic trunk (BCT) that ascends above the sternal manubrium and crosses the pretracheal plane before dividing in the lower neck, the risk extends to anterior cervical dissection, thyroidectomy, and tracheostomy [10].

Cervical ribs, supernumerary osseous elements arising from the seventh cervical vertebra, are present in approximately 0.5–0.6% of the general population but are overrepresented among patients with thoracic outlet syndrome [8]. Even in the absence of neurovascular compression, cervical ribs alter lower-neck topography and modify the expected relationships between the vertebral arteries, subclavian arteries, and the anterior scalene muscle—relationships that matter during supraclavicular exposure and image-guided cervical interventions.

Vertebral artery morphogenesis depends on serial longitudinal anastomoses between the cervical intersegmental arteries. Disturbance of this process produces a spectrum of developmental variants including anomalous origin, absent segments, duplication, and atypical foraminal entry levels [13]. Vertebral artery agenesis, characterised by absence of the ostium, V1 segment, and variable proximal V2 extent without histological evidence of acquired occlusion, is the most extreme form of this spectrum and has been documented as an incidental CTA finding [5]. Functional reconstitution of the distal vertebral artery by a collateral vessel—most commonly the deep cervical artery or the ascending cervical artery—has been described in cases where the proximal vertebral supply is absent or severely compromised. Recognition of the reconstituted segment and its collateral source is essential before cervical instrumentation or endovascular navigation, because the posterior circulation may depend entirely on this route.

The course of the internal carotid artery in the parapharyngeal space is similarly variable. A retropharyngeal course of the internal carotid artery (ICA), in which the vessel approximates or contacts the posterior pharyngeal wall, has been systematically reviewed by Tudose et al. [14], who established a pooled prevalence and defined its procedural risk implications during nasotracheal intubation, transoral surgery, and pharyngeal approaches. Critically, Gupta et al. [7] documented that the ICA position in the retropharyngeal space can change between imaging studies, so a previously normal examination cannot confidently exclude the variant in a future procedure. The elongated styloid process shares this parapharyngeal risk context: both entities narrow the working corridor for transoral, pharyngeal, and tonsillar procedures, and their coexistence amplifies that risk.

Bilateral poor sternoclavicular joint (SCJ) articulation, defined as less than 25% of clavicular cortical surface in contact with the manubrium, was quantified as a normal variant in 4.8% of asymptomatic individuals by Tuscano et al. [15]. When bilateral, this variant transforms the suprasternal notch into an interclavicular space, altering the topography of the anterior approach to the neck and creating a potential window through which the left brachiocephalic vein (LBCV) may be projected.

It is reported a 76-year-old woman in whom CTA revealed the concurrent presence of a common brachiocephalic–left common carotid origin, bilateral rudimentary cervical ribs, a high-riding pretracheal BCT, a right-sided proximal carotid loop, bilateral common carotid tortuosity, a retropharyngeal/retrotonsillar loop of the right ICA, a medial loop of the right external carotid artery at the hyoid greater horn, left vertebral artery segmental agenesis reconstituted by a tortuous deep cervical artery, bilaterally elongated styloid processes, and bilateral poor SCJ articulations with the LBCV projecting into the interclavicular space. The combination of these variants creates a distinctive surgical anatomy relevant to anterior neck, thyroid, tracheostomy, supraclavicular, pharyngeal, and endovascular approaches.

## Materials and methods

Archived computed tomography angiography (CTA) files of a 76-year-old woman were studied anatomically, with multiplanar reformatting and three-dimensional reconstruction. The examination had been performed to exclude a suspected neurosurgical pathology, which was not confirmed; the patient had no documented thoracic-outlet, dysphagic, airway, or Eagle-syndrome–related complaints, and all variants reported here were incidental. The study had been acquired on a 64-slice Siemens SOMATOM Definition AS scanner (Siemens Healthineers, Forchheim, Germany) at 120 kVp with automatic tube-current modulation (CARE Dose4D; 150–200 reference mAs), a 64 × 0.6 mm detector configuration with z-flying focal spot, gantry rotation time 0.5 s, and pitch 0.9. Iodinated contrast medium (iohexol 350 mg I/mL, 70 mL) was injected intravenously at 4 mL/s followed by a 30 mL saline chaser, with bolus tracking in the ascending aorta (threshold 100 HU). Axial images were reconstructed at 0.75 mm slice thickness with 0.5 mm increment using a medium-smooth vascular kernel (B26f). Source images were post-processed for multiplanar reformation (MPR), maximum-intensity projection, and three-dimensional volume-rendered reconstruction. All segments were assessed on MPR in three orthogonal planes; in particular, the expected course of the left V1 and proximal V2 vertebral segments was specifically interrogated on thin-section MPR to confirm true segmental absence rather than a string-like hypoplastic or occluded lumen. All measurements were performed directly on the DICOM image by the senior researcher (M.C.R.). As a retrospective study of archived, de-identified imaging, the work was conducted in accordance with the Declaration of Helsinki.

## Results

CTA showed combined osseous and arterial variants involving the aortic arch, supraaortic trunks, carotid arteries, vertebral arteries, styloid processes, and SCJs. The CTA had been performed to exclude a suspected neurosurgical pathology, which was not confirmed; the variants reported here were incidental findings on an otherwise negative study.

Bilateral rudimentary cervical ribs were found. Both were incomplete cervical ribs that tapered laterally without a distal bony or articular connection to the first thoracic rib (incomplete type; Gruber type I–II), arising from the C7 transverse processes.

**Fig. 1:**
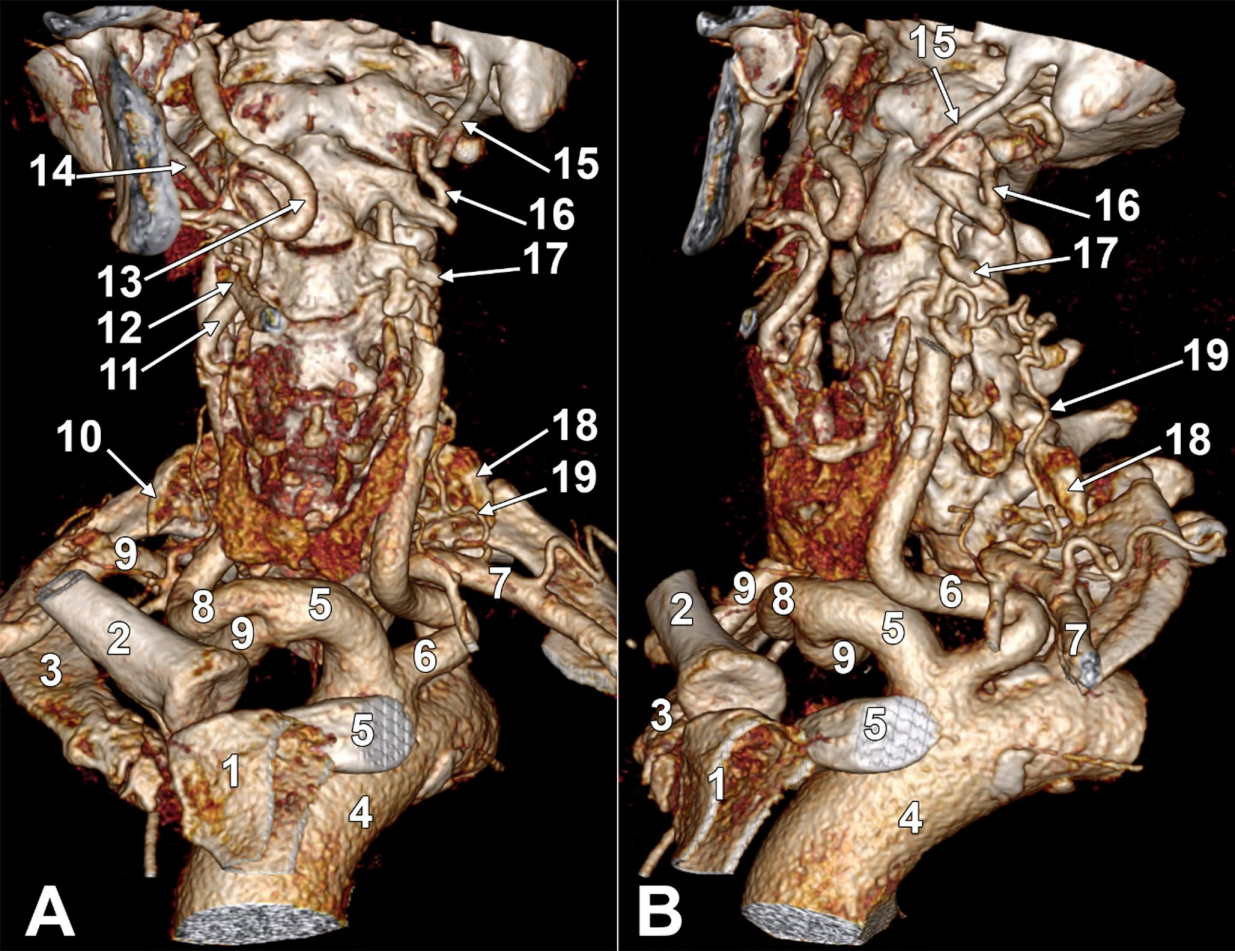
Multiple arterial variations depicted by three-dimensional volume renderings. **A** Anterior view. **B** Left lateral view. (1) sternal manubrium; (2) right clavicle; (3) first right rib; (4) aortic arch; (5) brachiocephalic trunk; (6) left common carotid artery; (7) left subclavian artery; (8) right common carotid artery; (9) right subclavian artery; (10) right cervical rib; 11. right external carotid artery; 12. greater hyoid horn; 13. retrotonsillar loop of the right internal carotid artery; 14. right styloid process; 15. left styloid process; 16. distal part of the V2 segment of the left vertebral artery; 17. C3 transverse process; 18. left cervical rib; 19. left deep cervical artery (joins the distal V2 and compensates the aplastic proximal V1 segment)

CTA demonstrated a common origin of the BCT and left common carotid artery, a common brachiocephalic–left common carotid origin (so-called bovine aortic arch). The BCT was high riding at 2.11 cm above the sternal manubrium. It reached immediately inferior to the thyroid isthmus at the level of C7, crossed the trachea from left to right in a pretracheal course, and divided in the lower neck, inferior to the right thyroid lobe, into the right common carotid artery and right subclavian artery (Fig. 1).

The right common carotid artery had marked proximal tortuosity. After its origin, it formed an initial rightward loop above the right subclavian artery. Its subsequent vertical turn lay 0.55 cm anteromedial to the right vertebral artery origin (Fig. 1).

**Fig. 2:**
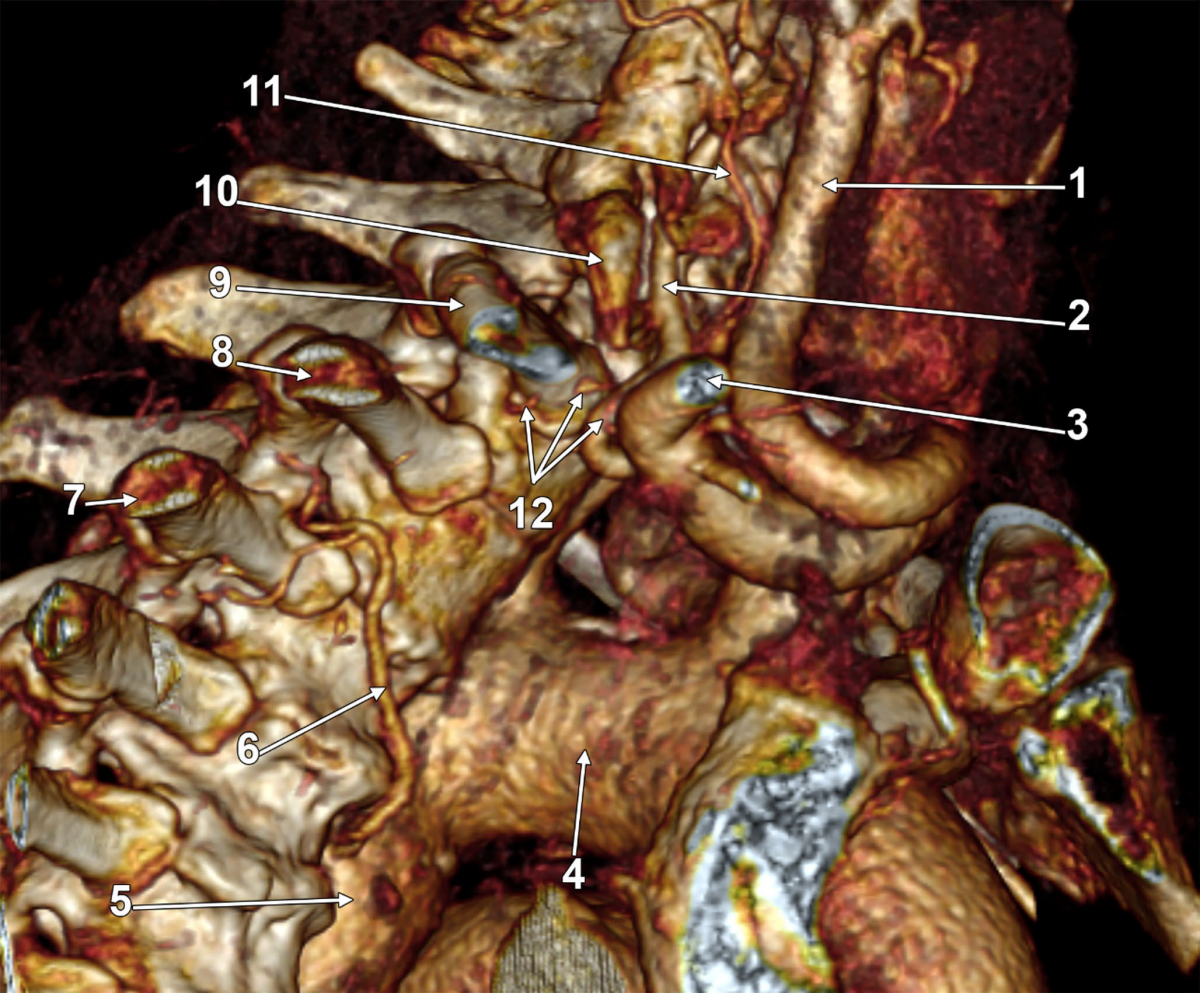
Lateral view of the upper arteries of the right intercostal spaces. Three-dimensional volume rendering, right lateral view. (1) common carotid artery; (2) vertebral artery; (3) subclavian artery; (4) aortic arch; (5) thoracic aorta; (6) common posterior intercostal trunk; (7) third rib; (8) second rib; (9) first rib; (10) cervical rib; 11. deep cervical artery; 12. superior intercostal artery

The right vertebral artery arose from the posterior wall of the right subclavian artery, 1.07 cm anteroinferior to the neck of the first rib. Its V1 segment had a luminal calibre of 0.29 cm, ascended anterior to the right cervical rib, and entered the C6 transverse foramen. The right deep cervical artery arose from the superior aspect of the right subclavian artery 0.5 cm distal to the right vertebral artery origin, coursed lateral to the carotid tubercle (Chassaignac tubercle), and then continued deep to the C5 transverse process. The right superior intercostal artery arose from the subclavian artery at 0.93 cm distally to the deep cervical artery, but it supplied only the first right intercostal space. The second and third right intercostal space were supplied by a common trunk that left the thoracic aorta at the level of the T3-T4 disc (Fig. 2).

On the left, the common carotid artery formed a large initial posterolateral loop measuring 2.12 cm immediately above the aortic arch and anterior to the thoracic segment of the left subclavian artery; distally to this loop, it continued cranially in the neck. The left vertebral artery lacked an ostium, V1 segment, and proximal V2 segment, a pattern consistent with segmental agenesis. No arterial stump, intraluminal calcified or soft plaque, mural thrombus, or luminal tapering was identified at the expected proximal vertebral course, and the C6–C5 transverse foramina on the left were correspondingly diminutive; on thin-section multiplanar reformation no string-like residual lumen was demonstrable along the expected V1/proximal-V2 course, supporting true segmental absence rather than a chronically occluded but present vessel. These features favour a developmental (congenital) absence over an acquired proximal occlusion. The left superior intercostal artery arose independently from the posterior aspect of the left subclavian artery. At 0.84 cm distal to this origin, the left deep cervical artery arose from the posterior side of the left subclavian artery, ascended anterior to the left cervical rib, and continued tortuously posterior to the C6-C4 transverse processes. In the intertransverse space at the C3–C4 intervertebral disc level, it joined the inferior end of a patent distal V2 vertebral artery segment measuring 0.28 cm in luminal diameter. Distal to this reconstitution point, the left vertebral artery followed the expected cervical and intracranial course and terminated in the posterior cranial fossa as a PICA-type vertebral artery with bilateral cerebellar distribution (Fig. 1). The intracranial vasculature, which lay outside the scope of this cervical variant report, was reviewed and confirmed that the study was adequately indicated; its detailed description is not reported here.

Additional right carotid variation was present. The right external carotid artery formed a medial loop on the inner side of the greater horn of the hyoid bone. The right internal carotid artery formed a large medial retropharyngeal/retrotonsillar loop, 1.56 cm deep, anterior to the axis (Fig. 1).

The styloid processes were bilaterally elongated and continuous, measuring 3.67 cm on the right and 3.93 cm on the left (Fig. 1).

**Fig. 3:**
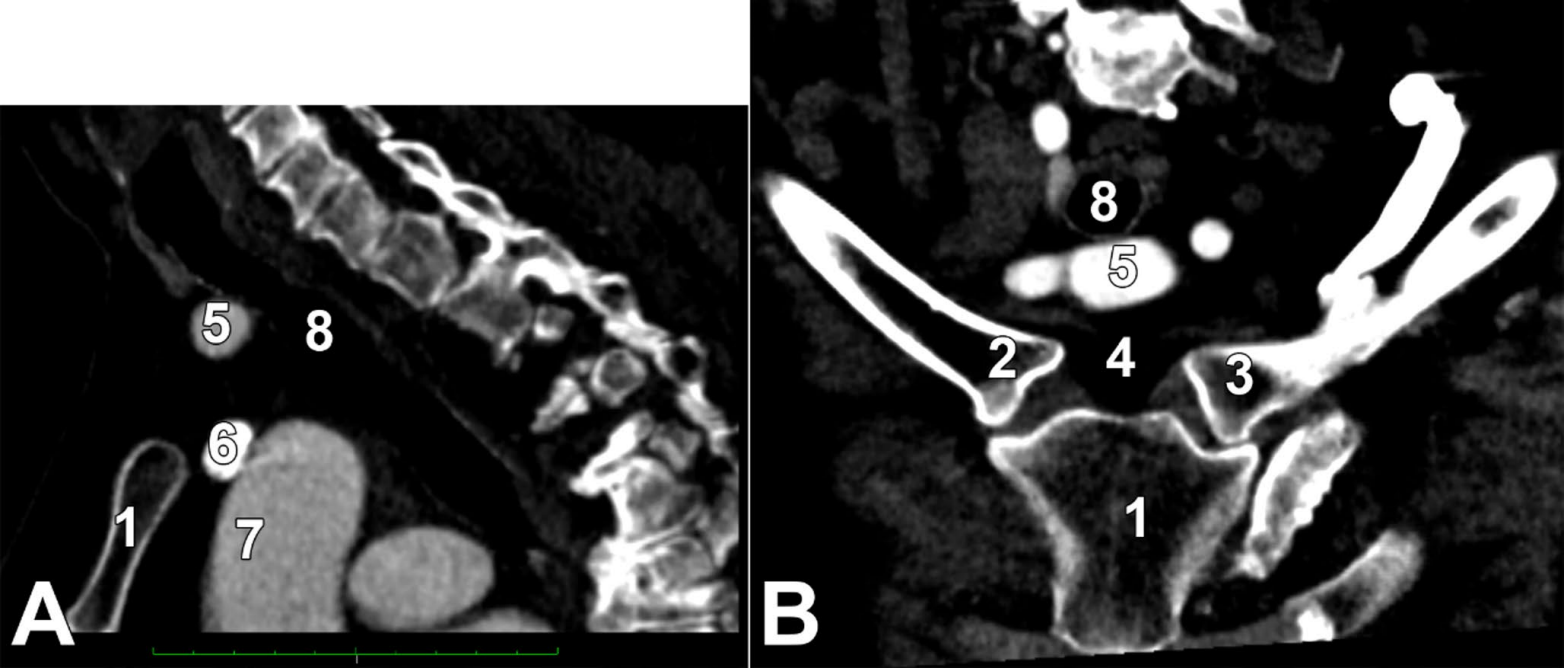
The suprasternal interclavicular space. **A** Sagittal slice, left view. **B** Coronal slice, anterior view. (1) sternal manubrium; (2) sternal end of the right clavicle; (3) sternal end of the left clavicle; (4) suprasternal interclavicular space; (5) brachiocephalic trunk; (6) left brachiocephalic vein; (7) aorta; (8) trachea

Poor SCJ articulations converted the suprasternal notch into an interclavicular space in which was projected the left brachiocephalic vein (LBCV) (Fig. 3). The clavicular cortical surface in contact with the manubrium measured 0.47 cm of a total articular width of 2.35 cm on the right (approximately 20%) and 0.5 cm of 2.4 cm on the left (approximately 21%); both values fall below the 25% threshold used to define poor SCJ articulation, confirming the diagnosis quantitatively and bilaterally.

## Discussion

This case is characterised by the simultaneous occurrence of anatomical variants spanning the aortic arch, supraaortic trunks, carotid arteries, vertebral arteries, styloid processes, and sternoclavicular joints, rather than by a single isolated anomaly. The common origin of the brachiocephalic trunk and left common carotid artery—the so-called bovine aortic arch, a colloquial misnomer for this human pattern—is the most prevalent human aortic-arch branching variant and carries well-recognised medico-surgical implications, particularly where supraaortic geometry must be assessed before open or endovascular procedures [11]. In the present case, this common origin coexisted with a BCT that rose 2.11 cm above the manubrium, crossed the trachea from left to right in a pretracheal plane, and bifurcated below the right thyroid lobe at the level of C7. This configuration creates high-risk anatomy for tracheostomy, low-neck dissection, thyroid surgery, and every anterior cervical approach that traverses the suprasternal region [10].

Projection of the left brachiocephalic vein (LBCV) into the interclavicular space represents a topographic extreme of normal positional variability rather than a discrete named anomaly, and in the present case it arose directly from bilateral poor sternoclavicular joint articulation converting the suprasternal notch into an open interclavicular window. In the largest CT-based prevalence study of major vessels anterior to the cervical trachea, Weightman and Gibbs [16] screened 500 consecutive thoracic CT examinations and found that 264 scans (53%) demonstrated at least part of a major vessel in the suprasternal notch, with the LBCV among the contributing structures lying directly anterior to the trachea in that region. The strongest predictor of this position was the length of trachea above the manubrium: patients with a manubrio-cricoid distance exceeding 50 mm had a 93% probability of a major vessel in the suprasternal notch [16]. A directly comparable cadaveric case was described by Budhiraja and Rastogi [3], in which the LBCV crossed anterior to the trachea at the level of the thyroid gland, placing it squarely in the path of the standard midline incision for open or percutaneous dilatational tracheostomy; the authors emphasised that haemorrhage from an unrecognised high-riding LBCV could be fatal, because venous laceration at this calibre retracts into the mediastinum and cannot be controlled through a cervical wound alone. An interclavicular LBCV projection is therefore an incidental CTA finding with direct relevance to tracheostomy, thyroidectomy, and mediastinoscopy whenever the anterior approach traverses the suprasternal region.

In standard anatomy, the costocervical trunk arises from the subclavian artery and divides into the superior intercostal artery and the deep cervical artery. The superior intercostal artery in turn supplies the first and second posterior intercostal spaces [6].

Bergman et al. [2] systematically documented the variational anatomy of the costocervical trunk across 611 sides from laboratory specimens. A discrete costocervical trunk was absent in 4.91% of sides: in 3.92% (24 sides) both the superior intercostal and deep cervical arteries arose directly and independently from the subclavian artery, while in a further 0.98% the deep cervical artery left the subclavian artery separately and the superior intercostal artery arose from the internal thoracic artery instead. The right-sided pattern in the present case—with the superior intercostal and deep cervical arteries arising independently from the subclavian artery, the superior intercostal artery supplying only the first intercostal space, and the second and third spaces fed by a common posterior intercostal trunk from the thoracic aorta—is consistent with the variational range documented by Bergman et al. and reflects the embryological flexibility of the intersegmental arterial system [2].

Bilateral rudimentary cervical ribs introduced an additional anatomical constraint at the thoracic outlet level. Although cervical ribs are found in fewer than 1% of the general population, they are substantially overrepresented among patients with thoracic outlet syndrome [8]. No overt compressive syndrome was identified on the present CTA, yet both ribs altered lower-neck topography in a functionally relevant way: the right vertebral artery ascended anterior to the right cervical rib before entering C6, and the left deep cervical artery similarly passed anterior to the left cervical rib before tracking posterior to the transverse processes to reconstitute the distal vertebral artery. These course modifications carry direct implications for supraclavicular exposure, thoracic outlet decompression, and image-guided lower-neck interventions.

The left vertebral artery pattern is the most distinctive vascular finding in this case. Complete absence of the ostium, V1 segment, and proximal V2 segment, without CTA evidence of luminal occlusion or mural thrombus, strongly favours a developmental mechanism over acquired proximal obstruction. Vertebral artery formation depends on sequential longitudinal anastomoses between the cervical intersegmental arteries during embryogenesis; focal failure of this process can result in absent segments, anomalous origins, duplications, and non-standard foraminal entry levels [5, 13]. Reconstitution of the distal V2 segment via a tortuous deep cervical artery—ascending anterior to the left cervical rib and then tracking posterior to the transverse processes before joining the patent distal V2 at C4/3–C3—provides a well-defined collateral route, consistent with reported cases in which deep cervical channels sustain posterior-fossa perfusion when proximal vertebral supply is absent or critically reduced [9]. The PICA-type configuration of the distal left vertebral artery, with bilateral cerebellar distribution, means that the left vertebral territory distal to the reconstitution is supplied through this deep cervical collateral pathway, whereas the posterior circulation as a whole is not solely dependent on it, given the contribution of the contralateral vertebral artery. Loss of the deep cervical collateral would nonetheless jeopardise the left vertebral territory. Posterior-circulation mapping is therefore mandatory before any cervical instrumentation or endovascular navigation on the left side.

The principal diagnostic question raised by this segmental absence is whether it represents a developmental anomaly (agenesis) or an acquired proximal occlusion, because the two have different clinical implications. Several imaging features in the present case favour a developmental mechanism. First, the vertebral ostium itself was absent, with no arterial stump arising from the subclavian artery; an acquired chronic occlusion typically leaves a recognisable origin or tapering remnant. Second, no calcified or non-calcified atherosclerotic plaque, intraluminal thrombus, dissection flap, or surrounding inflammatory mural change was identified along the expected proximal course, arguing against atherosclerotic occlusion, dissection, post-traumatic obstruction, or a large-vessel arteritis such as Takayasu disease. Third, the corresponding transverse foramina were diminutive on the affected side, a finding that accompanies congenital absence of the segment rather than acquired loss of a previously normal vessel. Fourth, the reconstituted distal segment was of normal calibre and smoothly continuous with the collateral, without the irregular re-canalisation appearance of organised thrombus. A specific alternative to consider, as the deep cervical artery can enlarge to compensate when a vertebral artery is occluded rather than absent, is a hyperplastic deep cervical collateral bridging a chronic proximal vertebral occlusion; we regard this as unlikely here because thin-section multiplanar reformation showed no residual proximal vertebral lumen, stump, or occlusive plaque and the ipsilateral transverse foramina were hypoplastic, a combination that accompanies developmental absence rather than acquired occlusion of a previously formed vessel. Taken together, these features support segmental agenesis with deep cervical collateralisation. We nonetheless acknowledge that, in the absence of catheter angiography or histological confirmation, an old, fully remodelled acquired occlusion cannot be excluded with absolute certainty; this is stated as a limitation below.

A further feature of this case is the widespread arterial looping and tortuosity affecting both common carotid arteries, the right external and internal carotid arteries, and the supraaortic trunks. Such looping is characteristic of the ageing arterial wall: progressive fragmentation and disorganisation of medial elastin, collagen remodelling, smooth-muscle loss, and the elongation that accompanies long-standing systemic hypertension cause arteries to buckle within their fixed anatomical compartments, producing kinks, coils, and redundant loops. These geometric changes are not merely morphological. Looping and kinking introduce abrupt changes in flow direction that generate flow separation, recirculation zones, and locally disturbed wall shear stress; these haemodynamic disturbances promote endothelial dysfunction, predispose to mural degeneration and, in susceptible vessels, to dissection or thrombosis, and may reduce distal perfusion reserve when a loop is acutely angulated. In an elderly patient in whom several major arteries are simultaneously tortuous, the cumulative effect is both a technical obstacle to catheter navigation and a theoretical contributor to regional flow instability, reinforcing the value of detailed preprocedural three-dimensional vascular mapping.

The carotid findings compound the operative risk in the upper neck and pharyngeal region. Both common carotid arteries were proximally tortuous; the right external carotid artery formed a medial loop on the inner surface of the hyoid greater horn; and the right internal carotid artery described a large 1.56-cm retropharyngeal and retrotonsillar loop anterior to the axis. When the ICA follows a retropharyngeal course it enters the procedural corridors used for transoral, pharyngeal, and tonsillar surgery, and may be lacerated during airway manipulation or retropharyngeal dissection [7, 14]. Because retropharyngeal carotid position can change between imaging studies, a single normal CTA does not definitively exclude the variant at the time of a subsequent procedure [7]. Structured CTA reporting for operative planning should therefore systematically describe the spatial relationship of all carotid loops to the pharyngeal wall, hyoid bone, thyroid gland, and cervical vertebrae.

The bilaterally elongated styloid processes—3.67 cm on the right and 3.93 cm on the left—belong to the same parapharyngeal risk cluster as the retropharyngeal ICA loop. Both processes were continuous, without pseudoarthroses. CT-based studies from the same institutional group, including morphometric CBCT work on styloid process anatomy and CTA characterisation of the external carotid artery–styloid process relationship, have established that elongated continuous styloid processes narrow the parapharyngeal working corridor and may contact adjacent carotid segments [1, 4]. When styloid elongation coexists with a tortuous or retropharyngeal ICA—as in the present case—the two variants synergistically reduce the parapharyngeal working corridor and jointly increase the risk of vascular injury during transoral, pharyngeal, or tonsillar procedures. Even in the absence of Eagle syndrome symptoms, this combination warrants explicit reporting because styloid-related carotid dissection has been documented [17].

This CTA therefore documents a multilevel anatomical configuration with compounding implications for surgical planning and radiological interpretation. The individual variants—common brachiocephalic–left common carotid origin, high-riding BCT, bilateral cervical ribs, deep cervical–vertebral collateralisation, retropharyngeal ICA loop, elongated styloid processes, and interclavicular LBCV projection—should not be reported as isolated incidental findings; together, they reconfigure operative corridors from the superior mediastinum to the upper cervical spine and parapharyngeal space. Preoperative multiplanar and three-dimensional CTA review is therefore advisable before any low-neck, thyroid, tracheostomy, transoral, pharyngeal, carotid, or endovascular procedure in patients with a comparable cervicothoracic variant phenotype.

The principal limitation of this report is its morphological scope: CTA characterises vessel geometry but cannot establish symptom causality, quantify dynamic haemodynamic effects, or prove that any variant is currently producing compression or ischaemia. In addition, neither catheter (digital subtraction) angiography nor histological examination was available; the developmental nature of the left vertebral segmental absence is therefore inferred from the supportive CTA criteria described above, and a fully remodeled chronic acquired occlusion cannot be excluded with absolute certainty. Nevertheless, the documented co-occurrence of these findings in a single patient constitutes a clinically informative example of cervicothoracic variant clustering and reinforces the value of systematic, patient-specific preprocedural vascular mapping.

To our knowledge, the present CTA documents an unusual cervicothoracic variant cluster in which a common brachiocephalic–left common carotid origin and high-riding pretracheal brachiocephalic trunk coexist with bilateral cervical ribs, segmental left vertebral artery agenesis reconstituted by a tortuous deep cervical collateral pathway, parapharyngeal carotid looping, elongated styloid processes, and poor sternoclavicular articulation with interclavicular projection of the left brachiocephalic vein. The novelty lies in the interaction of these variants across several operative corridors rather than in any single isolated anomaly.

## Data Availability

The imaging data analysed in this report are not publicly available because they derive from archived clinical CTA examinations and contain potentially sensitive patient-related information. De-identified data relevant to the anatomical findings may be made available by the corresponding author upon reasonable request, subject to institutional approval and applicable privacy regulations.

## References

[1] Andrei F, Motoc AG, Didilescu AC, Rusu MC (2013) A 3D cone beam computed tomography study of the styloid process of the temporal bone. Folia Morphol (Warsz) 72:29–35 23749708 10.5603/fm.2013.0005

[2] Bergman RA, Thompson SA, Afifi AK, Saadeh FA (1988) Compendium of human anatomic variation: text, atlas, and world literature. Urban & Schwarzenberg, Baltimore-Munich

[3] Budhiraja V, Rastogi R (2010) Unusual origin and potentially hazardous course of major blood vessels in neck. A clinically relevant rare case. Int J Anat Var 3:61–62

[4] Calota RN, Rusu MC, Vrapciu AD (2024) The external carotid artery and the styloid process. Curr Health Sci J 50:232–236. 10.12865/CHSJ.50.02.08 39380640 10.12865/CHSJ.50.02.08 PMC11459219

[5] Cuesta JP, Rodriguez LC, Bastidas N, Hernandez M (2022) Incidental finding of left vertebral artery agenesis: case report. Radiol Case Rep 17:4358–4361. 10.1016/j.radcr.2022.08.080 36188079 10.1016/j.radcr.2022.08.080 PMC9520501

[6] Gailloud P (2014) The supreme intercostal artery includes the last cervical intersegmental artery (C7): angiographic validation of the intersegmental nomenclature proposed by Dorcas Padget in 1954. Anat Rec (Hoboken) 297:810–818. 10.1002/ar.22893 24610867 10.1002/ar.22893

[7] Gupta A, Shah AD, Zhang Z, Phillips CD, Young RJ (2013) Variability in the position of the retropharyngeal internal carotid artery. Laryngoscope 123:401–403. 10.1002/lary.23393 22614949 10.1002/lary.23393 PMC4721920

[8] Henry BM, Vikse J, Sanna B, Taterra D, Gomulska M, Pekala PA, Tubbs RS, Tomaszewski KA (2018) Cervical rib prevalence and its association with thoracic outlet syndrome: a meta-analysis of 141 studies with surgical considerations. World Neurosurg 110:e965–e978. 10.1016/j.wneu.2017.11.148 29203316 10.1016/j.wneu.2017.11.148

[9] Mammen T, Vosoughi R, Wadhwa V (2013) Embolization of the deep cervical collaterals: a unique endovascular approach to prevent repeated posterior fossa strokes refractory to medical therapy. J Neurointerv Surg 5:e44. 10.1136/neurintsurg-2012-010363 23087382 10.1136/neurintsurg-2012-010363

[10] Moubayed SP, Ayad T (2014) High-riding innominate artery encountered during neck surgery. Otolaryngol Head Neck Surg 151:888–889. 10.1177/0194599814543771 25056601 10.1177/0194599814543771

[11] Rotundu A, Nedelcu AH, Tepordei RT, Moraru MC, Chiran DA, Oancea A, Mastaleru A, Costache AD, Chirica C, Grosu C, Mitu F, Leon MM (2024) Medical-surgical implications of branching variation of human aortic arch known as bovine aortic arch (BAA). J Pers Med. 10.3390/jpm14070678 39063932 10.3390/jpm14070678 PMC11278178

[12] Triantafyllou G, Piagkou M (2025) Is the term bovine aortic arch variant anatomically correct? Ann Vasc Surg 121:217. 10.1016/j.avsg.2025.06.018 40581177 10.1016/j.avsg.2025.06.018

[13] Tudose RC, Rusu MC, Hostiuc S (2023) The vertebral artery: a systematic review and a meta-analysis of the current literature. Diagnostics (Basel). 10.3390/diagnostics13122036 37370931 10.3390/diagnostics13122036 PMC10296927

[14] Tudose RC, Stan AD, Vrapciu AD, Munteanu IM, Dumitru CC, Triantafyllou G, Piagkou M, Rusu MC (2025) The retropharyngeal internal carotid artery: a systematic review, meta-analysis of the current literature, and evidence sampling. Clin Anat. 10.1002/ca.70072 41455123 10.1002/ca.70072

[15] Tuscano D, Banerjee S, Terk MR (2009) Variations in normal sternoclavicular joints; a retrospective study to quantify SCJ asymmetry. Skeletal Radiol 38:997–1001. 10.1007/s00256-009-0689-7 19308406 10.1007/s00256-009-0689-7

[16] Weightman WM, Gibbs NM (2018) Prevalence of major vessels anterior to the trachea at sites of potential front-of-neck emergency airway access in adults. Br J Anaesth 121:1166–1172. 10.1016/j.bja.2018.07.013 30336862 10.1016/j.bja.2018.07.013

[17] Zuber M, Meder JF, Mas JL (1999) Carotid artery dissection due to elongated styloid process. Neurology 53:1886–1887. 10.1212/WNL.53.8.1886 10563650 10.1212/wnl.53.8.1886

